# Efficiency of Near-Infrared Technology in the Clinical Detection of Carious Lesions: A Systematic Review

**DOI:** 10.1055/s-0043-1761187

**Published:** 2023-03-04

**Authors:** Ahmed Essam Elsawaf, Abdulsalam Youssef Al Deri, Patrick Samih Armanious, Abdallah Mahmoud Khasawneh, Ahmed Mahmood AlKhaja, Ahmad Ribhi Yasin, Natheer Hashim Al-Rawi, Sausan Al Kawas, Shishir Ram Shetty

**Affiliations:** 1 College of Dental Medicine, University of Sharjah, Sharjah, United Arab Emirates

**Keywords:** dental caries, near-infrared, radiographs, efficiency, detection, visual inspection, laser fluorescence, transillumination

## Abstract

The field of dentistry has seen various technological advances regarding caries detection, some lesions still prove to be difficult to detect. A reasonably new detection method, near-infrared (NIR), has shown good results in caries detection. This systematic review aims to compare NIR with conventional methods in terms of caries detection. Online databases (PubMed, Scopus, ScienceDirect, EBSCO, and ProQuest) were used for the literature search. The search was performed from January 2015 till December-2020. A total of 770 articles were selected, of that 17 articles qualified for the final analysis as per Preferred Reporting Items for Systematic Reviews and Meta-Analyses guidelines. The articles were assessed according to a modified Critical Appraisal Skills Programme checklist, and then synthesis of the review started. The inclusion criteria were clinical trials done in vivo on teeth with active caries of vital or nonvital teeth. This review excluded nonpeer reviewed articles, case reports, case series, opinions, abstracts, non-English written articles, studies of subjects with arrested caries, or teeth with developmental defects of tooth structure and teeth having environmental defects of tooth structure, as well as in-vitro studies. The review compared near-infrared technology with radiography, visual inspection, and laser fluorescence in terms of caries detection, sensitivity, specificity, and accuracy. The sensitivity of NIR ranged from 99.1 to 29.1%. Studies showed that NIR exhibited higher sensitivity for occlusal enamel and dentin caries. The specificity of NIR ranged from 94.1 to 20.0%. In enamel and dentinal occlusal caries, NIR demonstrated lower specificity than that of radiograph. The specificity of NIR in early proximal caries was low. Accuracy was determined in 5 out of 17 studies where the values ranged from 97.1 to 29.1%. The accuracy of NIR was the highest for dentinal occlusal caries. NIR shows promising evidence as an adjunct in caries examination due to its high sensitivity and specificity; however, more studies are required to determine its full potential in different situations.

## Introduction

Dental caries is one of the most prevalent dental diseases, caused by the demineralization of healthy tooth structure and linked to plaque, poor oral hygiene, and carbohydrate-rich diets. Due to late detection, subjectivity, and radiographic exposures, detecting caries using traditional procedures such as visual inspection (VI) and radiography (RG) can be difficult. 1 VI is the ability of the practitioner to correctly detect and identify carious lesions with a blunt probe and visual assessment of the tooth structure. Hence, a degree of skill and experience is a must. RG depends on the differential absorption of the penetrative ionizing radiation in the oral cavity. The ionising radiation is subsequently absorbed by the radiographic sensors forming radiographic images. Thus, the correct placement of the radiograph tube-head and sensor, machine settings, and skill of practitioner are a must. 1 2 3 4 As a result, many carious lesions go undetected, resulting in serious repercussions and a reduction in life quality. Therefore, several detection techniques, such as near-infrared technology (NIR), 2 3 4 have recently been created with the goal of increasing the overall accuracy of caries detection and improving the patient's dental health, an imaging technology that uses nonionizing radiation which is capable of detecting the amount of mineralization of the tooth structure through measuring the difference in the amount of scattering and absorption of near-infrared light. 5 The differential reflection of light due to caries is then received by capturing devices and software which produce a picture that shows carious cavities clearly, thus aiding in the detection of caries. 6

Various articles have recently examined alternative techniques for detecting dental caries in proximal and occlusal surfaces, including near infrared. 6 7 NIR has also been compared with laser fluorescence (LF) and VI in other research. 8 In addition, multiple papers had conflicting results regarding the overall performance of NIR with some showing a high rate of caries detection, while others showing a lower detection capability of NIR. 2 3 4 5 Subsequently, there is a need to determine whether NIR yields more accurate and appropriate detection capabilities for carious lesions compared with conventional methods of caries detection. The goal of this systematic review is to see if NIR can provide more accurate and suitable detection results than traditional caries detection and assessment approaches.

## Methods

Protocol registration—This systematic review was conducted with the standard regulations of Preferred Reporting Items for Systematic Reviews and Meta-Analysis statement. It is registered with the appropriate guideline protocol with FigShare (10.6084/m9.figshare.20055287).

Focused question—Is NIR a more accurate and suitable detection technology of carious lesions than traditional techniques of detection and assessment?

Search strategy—Both online and manual browsing were used as search strategies to extract the literature. The databases used were PubMed, Scopus, ScienceDirect, EBSCO, and ProQuest. The literature search was performed from January 2015 up to and including December 2020 to identify the relevant literature. The keywords used for the database searching was: “Near-infrared spectroscopy” AND “Demineralization,” “Near-infrared spectroscopy” AND “Dental caries,” “Caries” AND “NIRS,” “NIRS” AND “Demineralization,” “Dental caries” AND “NIR,” “Caries examination” AND “NIR,” “clinical.”

Eligibility criteria (inclusion/exclusion criteria)—In this systematic review, the article selection was restricted to controlled clinical trials. The case selection was strictly limited to in vivo studies with patients having carious lesions, whether incipient, primary, or secondary caries. Studies of patients with carious lesions in vital or nonvital teeth were also included. Only full-text articles were considered. This study did not include nonpeer reviewed articles, case reports, case series, opinions, brief communications, abstracts, theses, non-English written articles, or studies involving patients with arrested caries, developmental defects in tooth structure, or environmental effects on tooth structure development, or in-vitro studies.

Data extraction—Six investigators (split into three pairs) had gone over the articles that had been obtained individually. This procedure was completed in four phases. The first step was to use the provided search strategy to search the selected databases. The findings were tabulated in Microsoft Excel in the second phase of data extraction, followed by the elimination of duplicates. The third stage involved fine-tuning the number of results based on the eligibility criteria and the screening of article titles and abstracts. Finally, the full-text articles were evaluated for eligibility, and the qualitative synthesis was conducted using the remaining articles. The articles were only included if the investigators agreed on the ones to include. If the pairs of investigators could not agree, an expert reviewer (S.R.S.) was consulted for a third, unbiased view.

**Fig. 1:**
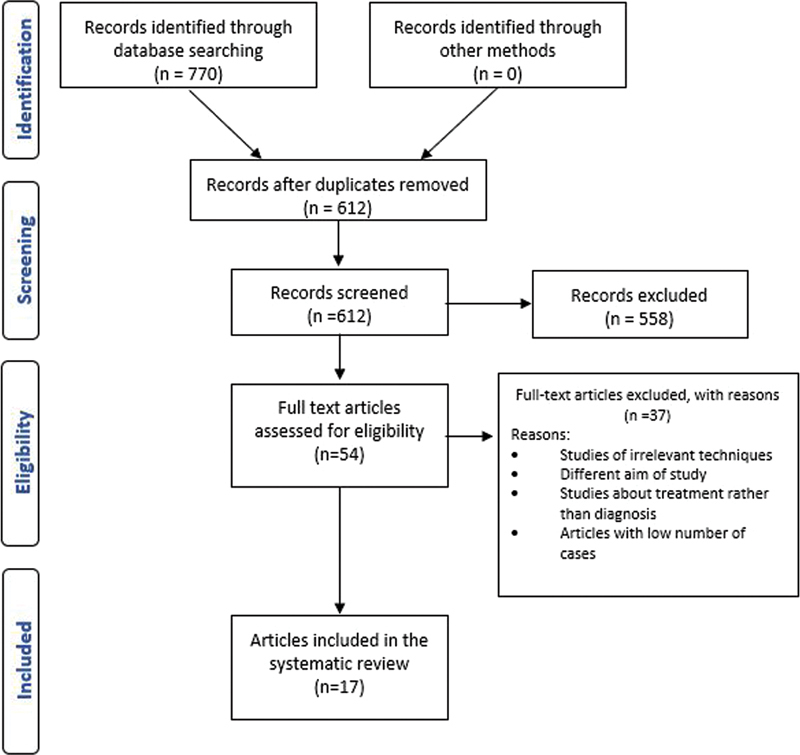
PRISMA flowchart of the systematic review.

Study selection—Through online database scanning, a total of 770 articles were identified. A total of 85 duplicate articles were eliminated; hence, the number of studies was reduced to 612 records. The elimination of irrelevant papers resulted in 558 studies being excluded. The remaining 54 full-text articles were subjected to eligibility testing, with 37 being rejected because they did not meet the criteria as shown in ( Fig. 1 ).

**Fig. 2:**
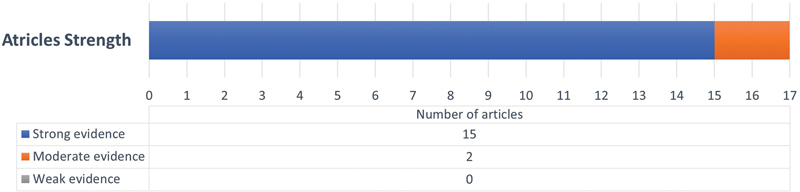
Strength of evidence of the selected studies.

Studies quality assessment—According to the modified Critical Appraisal Skills Programme checklist for clinical research, the quality of the included studies and the risk of bias were assessed. 9 The proportion of yes/no answers were used to assess the overall strength of the evidence. Strong evidence was defined as a proportion of more than 66%, moderate evidence was defined as 33 to 66%, and poor evidence was defined as less than 33%. Out of the 17 articles assessed, 15 were considered with strong evidence, 2 3 4 6 7 8 10 11 12 13 14 15 16 17 18 19 while 2 articles were of moderate strength (between 33 and 66%) 20 21 and no articles were considered of weak strength, as illustrated in ( Fig. 2 ; Table 1 ).

**Table 1:** Criteria for CASP checklist

1. Is the article/ research question relevant to the assignment?
2. Is the journal peer reviewed?
3. Is the article recently published (within 5 years) or is it seminal (i.e., an earlier article but which has strongly influenced later development)?
4. Was the hypothesis or research question clearly stated?
5. Was there a clear statement of what the investigators expect the study to find which can be tested, confirmed, or refuted?
6. Was the study design appropriate for the research question?
7. Did the methodology address important potential sources of bias?
8. Both inclusion and exclusion criteria described?
9. Were the statistical tests appropriate for the study design?
10. Was the sample size appropriate?
11. Was the data collected in a way that addressed the research question?
12. Did the research methods limit the influence of confounding variables?
13. Have ethical issues been taken into consideration? And approval has been sought from ethics committee?
14. Was the study performed according to the study methodology?
15. Were the statistical analyses performed correctly?
16. Is there evidence of statistical finding, i.e., changing statistical tests to ensure significance?
17. Was there a deviation from the planned protocol?
18. Did the results reliably support or refute the claims with high quality evidence?
19. Were the tables and figures appropriately used?
20. Was the original data presented clearly so that the readers can check the statistical significance of the results?
21. Do the results justify the conclusion?
22. Is there a clear statement of findings?
23. Is there adequate discussion of the evidence both for and against the researcher arguments?
24. Are the findings discussed in relation to the original research question?
25. What are the strengthens and weaknesses of the research article?
26. Assess the usefulness and clinical applicability of the study.
27. Does the study add anything new with evidence to the field of study?
28. Do the authors have any potential conflicts of interests, and have these been declared?
29. Who has funded this study and can we trust their objectivity?
30. Did the authors include relevant and important references?

Abbreviation: CASP, Critical Appraisal Skills Programme.

## Results

Study characteristics—The following are the characteristics of the 17 studies that met the criteria for the systematic review. There were a total of 1,301 participants in all of the investigations, which were all controlled clinical trials. Regarding the regions in which the studies were performed, seven studies were performed in Middle East, and North Africa, 4 7 8 10 13 17 20 seven in Europe, 2 3 6 11 15 16 18 and three from United States. 14 19 21 ( Table 2 )

**Table 2:** Characteristics of the studies included in the systematic review

Title of the study	Study design	Study aims and objectives	Author, Year	* N* , age, teeth, and surfaces examined	Diagnostic methods used	Magnitude of benefit	Quality of study	Main findings	Limitations and drawbacks
In vivo validation of near-infrared light transillumination for interproximal dentin caries detection	Controlled clinical trial	Investigating the diagnostic accuracy of NIR for proximal dentin caries detection and comparing it to established diagnostic methods	Kühnisch et al, 2015	* N* = 85, mean age = 25, permanent premolars and molars, proximal surfaces	NIR, radiograph s, laser fluorescence, visual inspection (VI)	Small	Strong evidence. (87%)	The accuracy of NIR achieved the same level as bitewings for the proximal dentin caries.	Selection bias was present, due to inclusion of lesions detected primarily on RG, and missing negative controls, limited the generalizability of their results.
Near-IR transillumination and reflectance imaging at 1,300 nm and 1,500–1,700 nm for in vivo caries detection	Controlled clinical trial	Assessing the performance of both NIR transillumination and NIR reflectance on teeth scheduled for extraction due to orthodontic treatment.	Simon et al, 2016	* N* = 40, age = 12–60, permanent premolars, occlusal and proximal surfaces	NIR, radiographs	Large	Strong evidence (68%)	NIR was significantly more sensitive than RG for the detection of lesions on both occlusal and proximal surfaces	Randomization statement is not mentioned.
A clinical study comparing digital radiography and near-infrared transillumination in caries detection	Controlled clinical trial	Compare the effective ness of detecting proximal carious lesions utilizing NIR system to tradition al RG.	Berg et al., 2017	* N* = 30, age ≥18, permanent premolars and molars, proximal surfaces	NIR, radiographs	Medium	Strong evidence (76.66%)	RG detected more sound surfaces or incipient enamel carious lesions compared with NIR, but fewer established lesions	Control group not established (sensitivity and specificity were not measured).
Clinical evaluation of near- infrared light transillumination in approximal dentin caries detection	Controlled clinical trial	Conventional caries detection techniques versus modern methods in detection of approximal dentin caries lesions.	Ozkan and Guzel, 2017	* N* = 157, age = 12–18, premolars and molar, proximal surfaces	NIR, radiographs, laser fluorescence and visual inspection (VI).	Large	Strong evidence (80%)	NIR showed almost equivalent efficiency to RG and better results compared with VI.	Enamel caries could not be validated. NILT images did not indicate the relationship between caries and dental pulp for the determination of caries extension.
Comparison of diagnostic methods for early interproximal caries detection with near- infrared light transillumination: an in vivo study	Controlled clinical trial	To evaluate the diagnostic capability of NIR and photostimulable phosphor bitewing radiograph (PSP-BWR) in detection of early interproximal carious lesion.	Baltacioglu and Orhan et al 2017	* N* = 26, mean age = 30.25, permanent premolars and molars, proximal surfaces	NIR, radiographs	Large	Strong evidence (87%)	NIR has an appropriate sensitivity and accuracy for detecting early interproximal caries lesions.	The usage of only two examiners may be a source of bias. Some carious lesions may have not been detected due to the scoring technique of used.
Digital transillumination in caries detection versus radiographic and clinical methods: an in- vivo study	Controlled clinical trial	Determination of agreement between NIR and clinical radiographic methods.	Lara-Capi et al., 2017	* N* = 52, age = 19–26, permanent premolars and molars, occlusal and proximal surface s	NIR, radiograph, visual Inspection (VI)	Medium	Strong evidence (83%)	NIR showed slightly higher sensitivity and lower specificity than RG methods in detection of early enamel lesions.	Method of double blindness was not mentioned. Bias could be due to the time limit present in the first NIR examination, but not in the second examination
Near-IR and CP-OCT imaging of suspected occlusal caries lesions	Controlled clinical trial	The aim of the study was to find a new sensitive imaging method for the detection of occlusal caries by comparing NIR and CP-OCT	Simon et al, 2017	* N* = 30, age = 18–60, permanent premolars and molars, occlusal surfaces	NIR, radiographs, visual inspection (VI)	Medium	Moderate evidence (63.6%)	Near-IR reflectance imaging at 1,500–1,700 nm yielded significantly higher contrast ( *p* < 0.05) of the demineralization in the occlusal grooves compare d with visible reflectance imaging.	No blinding was mentioned Randomization statement is not mentioned
A clinical study comparing digital radiography and near-infrared transillumination in caries detection	Controlled clinical trial	Compare the effective ness of detecting proximal carious lesions utilizing NIR system to tradition al RG.	Berg et al, 2018	* N* = 30, age ≥18, permanent premolars and molars, proximal surfaces	NIR, radiographs.	Medium	Strong evidence (76.66%)	RG detected more sound surfaces or incipient enamel carious lesions compared with NIR, but fewer established lesions	Control group not established (sensitivity and specificity were not measured).
Evaluation of occlusal caries detection and assessment by visual inspection, digital bitewing radiography and near-infrared light transillumination	Controlled clinical trial	Comparing the diagnostic outcome s of (VI), (BWR), and (NIR-LT, DIAGNO cam) for occlusal caries detection and assessment of posterior teeth.	Schaefer et al, 2018	* N* = 203, age ≥ 12, permanent premolars and molars, occlusal surfaces	NIR, radiographs, visual inspection (VI)	Medium	Strong evidence (73.33%)	VI detected the majority of occlusal caries. RG is indicated were insufficient fillings, deep carious lesions.	Sample might not be representative of all age groups. No reference standard.
Near infrared transillumination compare d with radiography to detect and monitor proximal caries: a clinical retrospective study	Controlled clinical trial	To compare near infrared transillumination device, DIAGNO cam (DC) and bitewing radiography (BW) for the detection of proximal caries.	Abdelaziz et al, 2018	* N* = 12, age = 2 2–32, permanent premolars and molars, proximal surfaces	NIR, radiographs	Large	Strong evidence (80%)	NIR is as reliable as RG to detect proximal dentin lesions. NIR detects proximal enamel lesions at an earlier stage than RG.	They neither implement blinding nor the double- blind method was implemented, and a few numbers of patients were participated in this study.
Proximal caries detection in permanent teeth by using DIAGNO cam: An in Vivo Study	Controlled clinical trial	The aim of this study was to compare the performance of NIR and RG in detection of dental caries.	Al Shaya et al, 2018	* N* = 374 proximal surfaces, age = NA, permanent premolars and molars, proximal surfaces	NIR, radiographs	Large	Moderate evidence (66.67%)	NIR was found to have similar accuracy to X-rays.	The inclusion criteria needed more explaining.
Accuracy of the DIAGNO cam and bitewing radiographs in the diagnosis of cavitated proximal carious lesions in primary molars	Controlled clinical trial	Evaluate the diagnostic abilities of NIR and RG in detecting cavitated proximal carious lesions in primary molars.	Alamoudi et al, 2019	* N* = 66, age 5– 8 years, primary molars, proximal surfaces	NIR, radiographs, visual inspection (VI)	Large	Strong evidence (76.66%)	NIR showed higher sensitivity and better accuracy than RG regarding the lesions reaching the dentin.	Sample might not be representative of all age groups.
Combined near-infrared light transillumination and direct digital radiography increases diagnostic in approximal caries	Controlled clinical trial	Evaluating the clinical ability of NIR for approximal dentinal caries detection in comparison to RG, and to assess their combination.	Melo et al, 2019	* N* = 88, age ≥ 18, premolars and molars, proximal surfaces	NIR, radiographs	Large	Strong evidence (78%)	NIR showed sensitivity similar to RG.	No randomization statements. No specific blinding mentioned
Clinical performance of clinical visual examination, digital bitewing radiography, laser fluorescence, and near- infrared light transillumination for detection of noncavitated proximal enamel and dentin caries	Controlled clinical trial	To compare the clinical performance of clinical-visual inspection using (ICDAS) II, digital BWR, ne (NIR-LT), and laser fluorescence (LF) for the detection of noncavitated proximal enamel and dentin caries.	Kocak and Cengiz-Yanardag, 2020	* N* = 335, ages = 12–18, permanent premolars and molars, proximal surfaces	NIR, radiographs, visual inspection (VI)	Large	Strong evidence (74%)	Digital bitewing radiography proved to be superior to all other caries diagnostic methods.	The usage of RG as the gold standard of the study may have been a source of bias.
In vivo correlation of near-infrared transillumination and visual inspection with bitewing radiography for the detection of interproximal caries in permanent and primary teeth	Controlled clinical trial	To compare the correlation between both NIR and visual Inspection with radiographs as a reference for the detection of clinically noncavitated interproximal caries in children.	De Zutter et al, 2020	* N* = 35, age = 4–16, permanent and primary molars and permanent premolars, proximal surfaces	NIR, radiographs, visual inspection (VI)	Medium	Strong evidence (87%)	NIR cannot be recommended as a single diagnostic tool for interproximal caries detection in primary teeth. NIR could be more accurate than BW radiography.	There was no randomization, and the double-blind method was not conducted. The absence of an absolute objective method as a reference diagnose is a source of bias in this study.
In vivo performance of near-infrared light transillumination for dentine proximal caries detection in permanent teeth	Controlled clinical trial	To compare diagnostic performance of NIR with visual ICDAS system, Bitewing radiograph (BWR), LED- based device, and laser fluorescence.	Dundar et al, 2020	* N* = 34, age = 22–55, permanent premolars and molars, proximal surfaces	NIR, radio graphic, laser fluorescence, visual inspection (VI).	Large	Strong evidence (77%)	The highest sensitivity values were recorded from Near-infrared Light Transillumination (NILTI) readings (99.1%), followed by BW scores (86.8%). The highest specificity values were recorded from ICDAS (100%).	Enamel caries was not verified in the study. No blinding was done. There was only one examiner.
Occlusal caries detection and diagnosis using visual ICDAS Criteria, Laser fluorescence measurements, and near-infrared light transillumination images	Controlled clinical trial	Compare the performance of visual inspection, laser fluorescence, and NIR in the detection of noncavitated occlusal caries lesions under clinical and laboratory conditions	Tassoker et al, 2020	* N* = 90, age = 18–25, third molars, occlusal surfaces	NIR, laser fluorescence, visual inspection (VI).	Large	Strong evidence (76.66%)	NIR is recommended in discriminating healthy and occlusal carious lesions. LF is a more sensitive method for discriminating enamel and dentine caries compared with the visual technique.	The inclusion of third molars is study was a limitation because of their variations. No descriptive figures or pictures were included.

Abbreviations: ICDAS, International Caries Detection and Assessment System; LED, light-emitting diode; NIR, near-infrared technology; RG, radiography.

Study outcome— Tables 3 and 4 illustrate the main findings of the included studies, and accordingly the following outcomes were found. As shown in Table 3 , NIR detected a significant quantity of caries in the occlusal surface, although less than VE. It also shown that it recognized more or the same lesions as RG for proximal early and late lesions( Table 3 ). Different caries detection methods were evaluated for sensitivity, specificity, and accuracy ( Table 4 ). The sensitivity of NIR was assessed in eight out of seventeen publications, with the greatest being 99.1% ^17^ and the lowest being 29.1%. 18 NIR was shown to have a greater sensitivity for occlusal enamel and dentin caries in studies. They also found conflicting outcomes for early and late proximal lesions, while the findings were equivalent to RG's. Specificity was measured in five of the seventeen trials, with results ranging from 94.1 ^17^ to 20.0%. 13 In the case of enamel and dentinal occlusal caries, NIR had a lower specificity than RG. NIR has a limited specificity in early proximal caries. However, the findings for late proximal caries were inconclusive. The accuracy of 5 out of 17 research was evaluated, with values ranging from 97.1 ^17^ to 29.1%. 18 The accuracy of NIR for occlusal enamel caries was unavailable, while it was the greatest for dentinal occlusal caries.

**Table 3:** Comparative analysis of outcomes of the studies using NIR and conventional methods

	Technique	*N*	Occlusal		Proximal		Statistical significance and outcome
			Enamel (Early)	Dentin (Advance d)	Enamel (Early)	Dentin *(* Advance d)	
Schaefer et al ^15^	NIR	3,174 a	* N* = 303	* N* = 5	–	–	VI detected the majority of occlusal carious lesions, followed by NIR
			(9.4%)	(0.2%)			
	RG		* N* = 5	* N* = 36	–	–	
			(0.2%)	(1.1%)			
	VI		* N* = 738	* N* = 4	–	–	
			(23%)	(0.1%)			
Lara-Capi et al ^3)^	NIR	2,496	* N* = 149 (17.9%)		* N* = 52	* N* = 31	Higher
		a			(3.1%)	(1.86%)	number of
	RG		–		* N* = 39	* N* = 31	early
					(2.3%)	(1.86%)	proximal
	VI		*N* = 152 (18.2%)		–	–	caries lesions were
							detected by
							NIR in
							comparison
							with BWRG.
							( *p* = 0.06)
Berg et al ^19^	NIR	67 b	–		* N* = 25	* N* = 42	Bitewing Radiography (BWXR)s
					(37.3 %)	(62.7%)	detected more early
	RG				* N* = 27 (40.3 %)	* N* = 33 (49%)	enamel lesion compared with near-infrared transillumination (NIRTI), but tended to detect fewer more established lesions ( *p* > 0.01)
Abdelaziz et al ^11^	NIR	139 a	–		* N* = 59	* N* = 48	NIR showed
					(42.4 %)	(34.5%)	better results in detecting
	RG				* N* = 46	* N* = 13	advanced
					(33.1	(9.3%)	interproximal
					%)		lesions compared
							with BW
							especially,
							and in the
							early enamel
							lesion.
Jablonski-momeni et al ^2^	NIR	193 a	–		* N* = 72	–	NIR was
					(37.3		found to have
					%)		similar result
	BWRG				* N* = 63 (32.6	–	to BWRG ( *p* = 0.07).
					%)		
	VI				* N* =	–	
					119		
					(61.6		
					%)		
Al Shaya et al ^20^	NIR	374 a	–		* N* =	* N* = 47	NIR was
					111 (29.6	(12.6%)	found to have similar
					%)		accuracy to
	RG				* N* = 52	* N* = 48	X-rays this
					(13.9	(12.8%)	was proved
					%)		by interrater reliability.

Abbreviations: NIR, near-infrared technology; RG, radiography; VI, visual inspection.

a Number of surfaces.

b Number of lesions.

**Table 4:** Comparative analysis of the parameters influencing the detection accuracy of NIR in relation to other conventional caries detection methods

	Technique	Sensitivity				Specificity				Accuracy			
		Occlusal		Proximal		Occlusal		Proximal		Occlusal		Proximal	
		E	D	E	D	E	D	E	D	E	D	E	D
Dündar et al ^17^	NIR	–			99.1%	–			94. 1%	–			97. 1%
	BWRG				86.8%				95. 6%				90. 2%
	VI				64.2%				100 %				78. 2%
	LF				81.1%				85. 3%				82. 8%
Kocak and Cengiz-Yanardag ^4^	NIR	–		86. 0%	57.0%	–				–		86 %	57. 0%
	BWRG			96. 0%	97%							96 %	97. 0%
	VI			98. 0%	15%							98 %	15. 0%
	LF			37. 0%	41%							37 %	41. 0%
Alamoudi et al ^7^	NIR	–			85.2%	–			56. 9%	–			
	BWRG				51.9%				57. 9%				
	VI				–				–				
Tassoker et al ^8^	NIR	–	93. 5%	–		–	69. 2%	–		–	90. 0%	–	
	VI		85. 7%				84. 6%				85. 5%		
	LF		80. 5%				76. 9%				89. 9%		
Simon et al ^21^	NIR	49. 0%	–	53. 0%	–	70. 0%	–	86. 0%	–	–			
	BWRG	1.0 %		23. 0%		100 %		96. 0%					
Melo et al ^16^	NIR	–			98.0%	–				–			
	BWRG				100%								
	VI				38.4%								
Ozkan and Guzel ^13^	NIR	–			82.0%	–		20.0%		–		80.0%	
	BWRG				83.0%			60.0%				82.0%	
	VI				54.0%			10.0%				56.0%	
	LF				60.0%			20.0%				59.0%	
Kühnisch et	NIR	–			D = 29. 1%	–		–		D = 29. 1%		–	
al ^18^					DEJ = 9 9.2%					DEJ = 9 9.2%			
	Bitewing radiography (BWRG)				96.1%					96.1%			
	VI				1.6%					1.6%			
	LF				66.7%					66.7%			

Abbreviations: D, dentin; E, enamel; LF, laser florescence; NIR, near-infrared technology; RG, radiography; VI, visual inspection.

Furthermore, NIR has been shown to have good detection accuracy for enamel and dentinal proximal cavities.

## Discussion

NIR's efficacy and accuracy, as well as its comparability to other standard caries detection methods, were assessed in many published publications. However, most traditional approaches have their own set of limitations and drawbacks. For example, VI's drawbacks include the capacity to detect lesions only if they are easy to reach, reliance on the examiner's subjective judgement, and inability to establish the real size of the lesion. 2 Furthermore, dental probes have the potential to damage the demineralized tooth surface and fissures, promoting the advancement of caries. 22 Due to these factors, RG has gained in popularity and usage in daily clinical practice, despite the dangers of ionizing radiation exposure and late detection of caries after the loss of 40% of the tooth structure. 20 Since there is variation in reflected light according to the degree of demineralization of the tooth, LF has been employed in conjunction with other methods as it reduces the need for RG while also giving an indication of the extent of the lesion.

This is possible since porphyrins produced by bacterial plaque lead to the fluorescence of the lesion after excitation by red light using a wavelength of 655 nm. 13 17 23 Despite promising scientific data in the literature, it was unable to outperform other traditional detection techniques in terms of accuracy. 4 24

As a result, the NIR is a relatively new caries detection method that has recently emerged on the scene. The basic principle is that, up to the time of this study, most dental equipment operated by producing NIR light with wavelengths spanning from 750 to 1,400 nm. 6 The light passes through the tooth structure, which reflects light differently according to the degree of demineralization, and this is visible as a difference in light value by the capturing device and software. 2 25 This is owing to the existence of water-filled pores with a high light scattering coefficient in the demineralized tooth structure. 26

According to the research, NIR has been shown to be effective in detecting caries on the occlusal and proximal surfaces of teeth, with good patient compliance. Furthermore, its ease of use, noninvasiveness, and lack of needless patient exposure encourage its usage in today's dentistry practice. 19 It can also distinguish between caries, stains, and hypomineralization of the tooth 2 27 because wavelengths greater than 1,300 nm avoid interreferences from stains, 28 and it can detect early and late cracks inside the tooth surface. 5 Even with all these advantages, NIR has certain limits. It was demonstrated that light rays cannot penetrate beyond the restorations; therefore, carious lesions behind big fillings and crowns cannot be seen. It is unable to detect the depth of caries and its relationship to the pulp, and it does not give an idea about periodontal health. 13 19 There is also the possibility that the vital pulp can alter normal light reflection, resulting in erroneous results. 27 29 Evidence addressing the detection of caries beneath restorations, on the contrary, is mixed, with some authors stating that NIR may detect caries under restorations, 21 while others refute this claim. 13

Because the advantages and limitations of each approach differed, 30 it was necessary to compare NIR to the other caries detection methods to find the best method overall. Accordingly, in Table 3 , the type of carious lesions was separated into occlusal caries and proximal caries, with further division into enamel (early lesions/International Caries Detection and Assessment System [ICDAS 0–2]) and dentin (late lesions extending to the DEJ and beyond/ICDAS 3– 5), to compare the number of carious lesions found using each technique by each study. However, the percentages of carious lesions found in the research cannot be compared since the number of samples ( *N* ) used in the studies differed, with some using the sample size as the number of tooth surfaces and others using the sample size as the number of carious lesions. As a result, the outcomes of each article were being compared.

Table 3 has demonstrated the following outcomes. In the case of occlusal caries, Schaefer et al discovered that VI recognized the majority of early lesions, followed by NIR, and finally RG. However, when compared with early lesions, all caries detection techniques found fewer late lesions. RG was the most common method of detection for advanced lesions, followed by NIR and VE, which both had similar results. 15 On the contrary, Lara-Capi et al did not find any significant differences between NIR and VI. 3 In line with these results, some controversies regarding the detection of occlusal caries were revealed. The number of lesions on the occlusal surfaces identified by NIR can be affected by the angle of light application, since different angles may not generate the same light reflection, 14 which could explain the discrepancies in the results between Lara-Capi et al and Schaefer et al. 3 15 Another reason for the discrepancy in the results is that the lesions in the research by Lara-Capi et al were not divided into enamel or dentin lesions due to the difficulties in detecting the depth of the lesions using NIR. 3

In terms of early proximal lesions, four articles 2 3 11 20 concluded that NIR detected more carious lesions than RG, However, VI was included in the study by Jablonski-Momeni et al, and it was found to be superior to all other techniques. On the contrary, Berg et al found that RG detected more lesions than NIR. 2 3 11 19 20 In the case of late proximal lesions, Berg et al and Abdelaziz et al found that NIR performed better than RG, while Al Shaya et al and Lara-Capi et al detected similar amounts of caries in both NIR and RG. 3 11 19 20 NIR, on the contrary, was found to be capable of detecting more or an equivalent number of lesions as RG for the detection of early and late proximal caries, according to the literature. An exception to that was a single study by Berg et al, which found that RG detected more sound surfaces or incipient enamel carious lesions compared with NIR and a fewer established lesions. In addition, only one study compared NIR with VI, which showed better results with VI. 2

As seen in Table 4 , the sensitivity, specificity, and accuracy of all the techniques were compared in terms of the tooth surface. The tooth surface was further divided according to the extent of caries into enamel (early) lesions or DEJ/dentin (late) lesions.

Simon et al was the only study which displayed occlusal early lesions; the results of this study showed that NIR had higher sensitivity but lower specificity than RG (sensitivity: 49.0 vs. 1.0%, specificity: 70.0 vs. 100%). 14

On the contrary, the occlusal late lesions were studied by Tassoker et al, which calculated the highest sensitivity values for NIR (93.5%) followed by VI (85.7%) and then LF (80.5%). However, it was evident in that study that the specificity of NIR was the lowest (69.2%) in comparison with LF (76.9%) and VI (84.6%). Tassoker et al also evaluated the accuracy and ended up with the highest accuracy results for NIR (90.0%) followed by LF (89.9%) and then VI (85.5%). 8

Two trials were included in this review for proximal early lesions. The first was performed by Kocak and Cengiz-Yanardag, 4 while the second was performed by Simon et al. When comparing the sensitivity of the NIR to that of the RG, both investigations disagreed. Kocak and Cengiz-Yanardag 4 determined that the highest sensitivity was found by VI (98.0%) and RG (96.0%), followed by NIR (86.0%), and finally LF (37.0%). However, Simon et al concluded that NIR had higher sensitivity (53.0%) than RG (23.0%). Simon et al determined that the specificity of RG (100%) is higher than that of NIR (70.0%), but did not calculate the accuracy. 4 14 In light of the previous table, it is clear that NIR has a high sensitivity but a low specificity when it comes to accurately detecting occlusal enamel caries. This is reinforced by the fact that the wavelength of the light utilized and the sensitivity of the software used allow for the reading and identification of early enamel demineralization without being influenced by dental variables like stains and coloring. 2 14 NIR also showed similar results when applied for the detection of occlusal lesions reaching dentin.

Finally, there was a wealth of information in the literature about the assessment of proximal dentinal caries lesions. Dündar et al 17 and Kühnisch et al 18 both found that the highest sensitivity values were achieved by NIR (99.1 and 99.2%, respectively), then RG (86.8 and 96.1%), followed by LF (81.1 and 66.7%), and finally VI (64.2 and 1.6%). Although Alamoudi et al 7 only looked at NIR and RG, they were able to support the data given by Dündar et al 17 and Kühnisch et al 18 because NIR had better sensitivity than RG (85.2 vs. 51.9%). However, Kocak and Cengiz-Yanardag 4 and Ozkan and Guzel 13 found that RG had the highest sensitivity (97.0 and 83.0%, respectively), followed by NIR (57.0 and 82.0%), then LF (41.0 and 60.0%), and finally VI (15.0 and 54.0%). This was also supported by the study done by Melo et al, 16 which claimed that RG had the highest sensitivity (100%), followed by NIR (98.0%), then VI (38.4%). As for the specificity, Ozkan and Guzel, 13 Alamoudi et al, 7 and Dündar et al, 17 all agreed that NIR had lower specificity than RG (20.0, 56.9, and 94.1% vs. 60.0, 57.9, and 95.6%). In studies including LF, Dündar et al 17 found that NIR had higher specificity than LF (94.1 vs. 85.3%), while Ozkan and Guzel 13 observed similar specificity for both NIR and LF (20.0% for both). NIR was evidently superior in terms of specificity to VI in the study done by Ozkan and Guzel, 13 (20 vs. 10%); however, Dündar et al 17 showed the highest specificity for VI (100%) in comparison with RG (95.6%), NIR (94.1%), and LF (85.3%). Finally, as for the accuracy, both Ozkan and Guzel 13 and Kocak and Cengiz-Yanardag 4 established that RG is the most accurate technique (82.0 and 97.0%), followed by NIR (80.0 and 57.0%), then LF (59.0 and 41.0%), and finally VI (56.0 and 15.0%); however, Dündar et al., 17 ended up with the highest accuracy values for NIR (97.1%), followed by RG (90.2%), then LF (82.2%), and finally VI (78.2%). The findings of using NIR for early and late proximal caries were mixed; nonetheless, it is clear that NIR has acceptable sensitivity, specificity, and accuracy. This is feasible because the NIR device's light may reach deeply hidden proximal caries, where it is reflected by the carious lesion and captured by a capturing device. However, in studies showing high sensitivity for NIR, a risk of overexposure was demonstrated. This could possibly lead to a drop in specificity, which explains the disagreements observed across studies addressing specificity. The overall accuracy of NIR was appropriate and comparable to that of RG, if not higher. 3 4 7 13 16 17 18

The use of NIR for occlusal and proximal caries proved to be simple, reliable, and repeatable, as evidenced by the literature's excellent inter- and intraexaminer reliability. NIR revealed a low level of subjectivity and a reduced demand for examiner experience. 6 10

The findings of such research lead the way for future dental clinical practice; therefore, it is critical to study them thoroughly and be aware of their limits. Because true diagnosis can only be made by histological studies, sources of limitation could exist in any clinical study determining the efficacy of in vivo detection of caries. However, because this is not possible, each study uses a different detection technique as a gold standard, which could be a source of bias. For instance, some studies use VI as a gold standard detection technique, others use RG, and some use a combination of techniques such as VI together with RG as the gold standard; thus, it is not possible to have a standardized gold standard among all the included articles. Another limitation is the presence of limited evidence within the literature regarding the use of NIR and occlusal caries. Because confirming the presence of the lesion necessitates opening the lesion, which may be considered unethical in modern dentistry practice, most publications avoid researching NIR and occlusal caries in vivo. As a result, data in the literature relating the use of NIR and occlusal caries are rare. Some of the studies included in the article had a small number of examiners, which might lead to bias. Furthermore, the variety of devices utilized in the research might potentially lead to bias, as it was difficult for all of the studies to employ identical RG, LF, and NIR equipment.

According to these findings, more research comparing NIR to other traditional caries detection techniques, particularly those looking at occlusal enamel caries, is needed. As a result, such technologies will have an evidence-based foundation that will encourage their use in everyday clinical practice.

Furthermore, because the literature on in vivo research is limited compared with that on in vitro studies, future studies evaluating NIR should follow well-developed study designs. Finally, further research is needed to compare the indications, benefits, and drawbacks of various NIR devices and their numerous wavelengths.

## Conclusion

Over the years, caries detection has been studied in detail. Operators have depended on multiple caries detection techniques which had various drawbacks and required skilled operators. However, NIR is a relatively new caries detection technique that may overcome many of the drawbacks of conventional techniques. According to this study, NIR has been carefully examined from the perspectives of numerous researchers, with its benefits and drawbacks clearly stated. NIR has been shown to be a potential complement to other traditional caries detection methods as it has high sensitivity, specificity, and accuracy in the detection of caries; nevertheless, NIR is a vast topic that requires further study in order for dental professionals to produce better and more accurate caries detection and progression approaches.
